# Anaemia among school children older than five years in the Volta Region of Ghana

**DOI:** 10.11694/pamj.supp.2014.17.1.3205

**Published:** 2014-01-18

**Authors:** Godfred Egbi, Matilda Steiner-Asiedu, Faribu Saalia Kwesi, Irene Ayi, Winfred Ofosu, Jacob Setorglo, Seth Selorm Klobodu, Margaret Armar-Klemesu

**Affiliations:** 1 Nutrition Department, Noguchi Memorial Institute for Medical Research, College of Health Sciences, University of Ghana, P. O. Box LG581, Legon, Ghana; 2 Department of Nutrition and Food Science, University of Ghana, P. O. Box LG 134, Legon, Ghana; 3 Parasitology Department, Noguchi Memorial Institute for Medical Research, College of Health Sciences, University of Ghana, P. O. Box LG581, Legon, Ghana; 4 Regional Health Directorate, Public Health Division, Ghana Health Services, P. O. Box 72 Ho, Volta Region, Ghana; 5 School of Medical Sciences, University of Cape Coast, PMB, Cape Coast, Ghana

**Keywords:** Anaemia, prevalence, school, children, malaria, parasitaemia, haemoglobin, ferritin, Ghana

## Abstract

**Introduction:**

Anaemia among children is a public health issue in Ghana. The Ghana School Feeding Programme (GSFP) was instituted on pilot basis in an effort to provide nutritious lunch to school children. Evidence on the nutritional status of pupils is needed to inform the expansion of GSFP. This study sought to assess anaemia among Ghanaian pupils.

**Methods:**

This cross-sectional study involved a random sample of 143 pupils aged 6 to 12 years. Blood samples were collected and analysed for serum-ferritin (SF), C-reactive protein (CRP), haemoglobin and malaria-parasitaemia (MP). Stool samples were examined for soil-transmitted helminthes. Dietary data were collected using the 24 hour-recall method on three non-consecutive days and a food frequency questionnaire. The Student's t-test was used to compare mean values between sexes. Binary logistic regression was performed to identify factors associated with anaemia. Statistical significance was set at p < 0.05

**Results:**

SF and haemoglobin concentrations were 23.9±15ng/ml and 120±11g/L respectively. The prevalence of anaemia was 30.8%. More females (41.5%) than males (21.8%) had anaemia (p < 0.005). Seventy-one percent of pupils had low SF levels. MP prevalence was 67.8%. Hookworm infestation was only observed in males (18.0%). Dietary iron and vitamin C intakes were 18.98±8.8mg and 23.7±6.7mg, respectively. Child's sex, SF and MP were associated with anaemia. Males had a lower likelihood of being anaemic (OR = 0.2, CI 0.1-0.5, p = 0.002)

**Conclusion:**

The study findings underscore the need for multi-pronged approaches that address both malaria control and nutrition in order to reduce anaemia among pupils.

## Introduction

Micronutrient deficiencies impose substantial health, economic, and social burdens worldwide [1]. Iron deficiency (ID) is the most prevalent haematologic disorder during childhood, globally [2]. One major consequence of ID is anaemia, which affects a large proportion of the world's population [3–7]. Anaemia has a range of adverse consequences including; poor cognitive performance, poor growth of infants, preschool and school-aged children; impairment of physical capacity and work performance of adolescents and adults; reduction in immune competence; and increased morbidity from infections in all age groups [8]. Severe anaemia is a common condition causing significant morbidity and mortality in children in Africa [9]. Severe anaemia carries a high “hidden” morbidity and mortality occurring in the months after initial diagnosis and treatment [9].

Anaemia in children is a public health issue in Ghana. It has been reported that 76% of Ghanaian children below five years of age [10], 73% of children aged 2-10 years [11], and 63% of school children aged 5-12 years suffer from anaemia [12]. Anaemia has multiple causes and associated risk factors which often work in tandem. They include various nutritional deficiencies, infections and infestations, as well as genetic defects including glucose-6-phosphate dehydrogenase (G6PD) deficiency, and haemoglobinopathies [13–15]. Studies in Kenya, India and Bangladesh have shown multiple coexisting causes of anaemia in individuals [16–18]. These causes include iron deficiency, vitamin A deficiency, malaria parasitaemia and other parasitic infestations. Traditionally, haemoglobin level has been used to estimate iron deficiency and iron deficiency anaemia even though haemoglobin estimates are neither specific nor sensitive as a screening test for iron [19].

Across the spectrum of anaemia, iron deficiency anaemia (IDA) is the most prevalent nutritional disorder worldwide accounting for about 75%-80% of the total burden of anaemia [4, 5]. IDA is partly induced by plant-based diets containing low bioavailable non-haeme iron [20]. Blood losses within the gastrointestinal tract due to intestinal parasites or inflammatory bowel disease may also contribute to IDA [20]. However, the major correlates of anaemia among Ghanaian children are largely understudied. Existing studies on anaemia focus primarily on preschool children (< 5 years) and little is known about anaemia in school-aged children in rural Ghana. The objective of the present study was to assess dietary intakes, iron status, and anaemia prevalence and its associated factors among Ghanaian school children aged 6-12 years. Study findings are expected to inform intervention strategies to reduce iron deficiency and anaemia among Ghanaian children. Method

## Methods

### Study setting

The study was conducted in the Kodzobi community of the Adaklu-Anyigbe district of the Volta Region of Ghana. Adaklu-Anyigbe is one of the newly created districts in the middle belt of the Volta Region. The study was cross-sectional in design and involved school children aged 6 to 12 years.

### Study sample

Using a power of 80% with a two-sided test significance level of 5% and mean difference in ferritin level and standard deviation from a recent work [21], a minimum sample size of 143 was estimated. To account for refusals (estimated at 13%) the desired sample size was increased to 162; 81 females and 81 males. A sampling frame of public basic schools not benefitting from the Ghana School Feeding Programme was constructed and Adaklu Kodzobi basic school was selected at random. Pupils in lower and upper primary as well as junior high school aged 6-12 years were randomly selected after stratification based on sex. Of 162 children approached, 143 provided assent and subsequently blood samples for biochemical analysis. Others refused citing religious and customary reasons.

### Ethical consideration

Ethical approval was obtained from the Institutional Review Board (IRB) of Noguchi Memorial Institute for Medical Research, College of Health Sciences, University of Ghana, Legon. Permission was obtained from the District Director of Education, the local chief and elders and the head of the school sampled. Written informed consent was also obtained from the parents/guardians of pupils.

### Procedures

Data were collected over a 4-week period in July 2010. Biological samples (blood and stool) were collected from each participant. Five millilitres of fasting venous blood were collected by a phlebotomist into eppendorf tubes without anticoagulants from a sub sample of participants (40 children in the morning of each day) before they had their breakfast meal. The blood sample collection was done in a private setting. Haemoglobin concentrations were determined immediately in the field using a Hemocue Hemoglobinometer (Hemocue AB, Angelhom, Sweden).The blood samples were transported on ice-chips to Noguchi Memorial Institute for Medical Research (NMIMR). Each blood sample was centrifuged at 3000g for 15 minutes in the laboratory and serum aliquots were prepared and stored at -80°C until they were analysed.

Serum ferritin levels were measured using the Enzyme-Linked Immunosorbent Assay Alpha Diagnostics Inc. (ADI), (ADI's Ferritin ELISA kit, Cat No. 1810, San Antonio, USA). Human C-Reactive Protein (CRP) levels, which indicate presence of inflammation, were determined with ADI's CRP ELISA kit (Cat No. 1000, San Antonio, USA). The age specific cut-off values for haemoglobin concentrations used to define anaemia were 115g/L for those aged 5-11 years and 120g/L for those aged 12 years [22]. Iron deficiency was defined as ferritin concentration ≤ 30ng/ml due to high prevalence of malaria [23]. Malaria parasitaemia was assessed using the Giemsa staining technique [24]. Soil-transmitted helminthes were identified in stool using the Standard Kato-Katz technique [24]. The stool and blood examinations were carried out at the Parasitology Department of NMIMR by a parasitologist. Dietary data were collected using the 24 hour-recall method on three non-consecutive days and a food frequency questionnaire [25, 26]. Dietary data were used to calculate (estimate) amount of nutrients consumed using Eish FPRO software Version 6.5 and the Ghana Food Composition Table [27].

### Data analysis

All data collected were analysed with SPSS (version 16.0). Variables were checked for normality. Haematological indices and dietary data were normally distributed so their summary values are presented as means plus standard deviations. The Mann-Whitney test was used to determine whether there was a significant difference between males and females for prevalence of low iron store and anaemia. Student's t-test was used to compare means of dietary data and haematological indices of sexes. Correlations between age, haemoglobin levels, ferritin levels, C-reactive protein concentrations and malaria parasitaemia were determined using the Pearson product-moment correlation test. Binary logistic regression was performed to identify factors associated with anaemia among the children. Statistical significance was set at p < 0.05 for all analyses.

## Results

The study comprised 65 females and 78 males aged 6-12 years. Participants’ mean age was 9.2±2.3 years. Twenty-eight percent (31% females and 26% males) of the participants were aged 12 years (Table 1).

**Table 1:** Background characteristics and biochemical indices of the study participants

Factor	Females (n = 65)	Males (n = 78)	P-value	Sexes combined (n = 143)
	(n = 65)	(n = 78)		(n = 143)
**Age distribution**	n(%)	n(%)		
6 years	13(20)	14(18)		27(19)
7 years	4(6)	11(14)		15(10)
8 years	9(14)	10(13)		19(13)
9 years	5(8)	8(10)		13(9)
10 years	5(8)	8(10)		13(9)
11 years	9(14)	7(9)		16(11)
12 years	20(31)	20(26)		40(28)
Serum ferritin concentration (ng/ml) mean ± SD	25.3 ± 16.3	22.6 ± 13.8	0.28	23.9 ± 15.0
Prevalence of low iron store (serum ferritin concentration <30ng/ml)) (%)	67.7	74.4	0.32	71.3
Haemoglobin concentration (g/L) mean ± SD	118 ± 9	121 ±12	0.9	120 ± 11
Prevalence of anaemia (haemoglobin concentration <120g/L) (%)	41.5	21.8	0.01*	30.8
CRP concentration (ng/ml) mean ± SD	44.6 ± 27.1	48.5 ± 29.0	0.51	46.7 ± 28.0
Prevalence of high CRP levels >10ng/ml (%)	100.0	91.8		95.7
Prevalence of malaria (%)	66.2	69.2		67.8
Prevalence of hookworm (%)		18.0		9.8

Cut off values: ferritin < 30ng/ml, haemoglobin < 115g/L for children below 12 years and 120g/L for children 12years and above; CRP = C-reactive protein. P-values obtained from independent t-test analysis.

* Significant at p < 0.05.

The mean serum ferritin concentration for both sexes was 23.9±15.0ng/ml. The mean serum ferritin concentration for females (25.3±16.3ng/ml) was higher than that for the males (22.6±13.8ng/ml). The mean haemoglobin concentration of the participants was 120±11g/L. No statistically significant difference was observed in the mean haemoglobin concentrations for females and males.

The prevalence of low iron store (serum ferritin concentration ≤ 30ng/ml) among the study participants was 71.3% (Table 2).

**Table 2:** Dietary nutrients intakes of the study participants

Factor	Females(n = 65)			Males (n = 78)			P-value
	Mean ± SD	NAR > 1 n(%)	NAR < 1 n(%)	Mean ± SD	NAR > 1 n (%)	NAR < 1 N (%)	
**Energy (kcal)**	939.50 ± 244.36	ND	ND	1018.61 ± 261.27	ND	ND	.06
**Protein (g)**	32.73 ± 10.46	59(91)	6(9)	31.12 ± 6.16	71(91)	7(9)	.26
**Fat (g)**	34.33 ± 7.53	ND	ND	32.83 ± 6.84	ND	ND	.22
**Iron (mg)**	19.49 ± 8.72	65(100)	-	18.55 ± 8.98	78(100)	-	.53
**Vitamin C (mg)**	23.0 ± 7.0	15(23)	50(77)	24.26 ± 6.54	22(28)	56(72)	.27

Nutrient Adequacy Ratio = NAR

RDA sources: ^1^Food and Nutrition Board, Institute of Medicine, National Academy of Sciences. Dietary Reference Intakes for Vitamin C, Vitamin E, Selenium, and Carotenoids (2000).

^2^Food and Nutrition Board, Institute of Medicine, National Academy of Sciences. Dietary Reference Intakes for Vitamin A, Vitamin K, Arsenic, Boron, Chromium, Copper, Iodine, Iron, Manganese, Molybdenum, Nickel, Silicon, Vanadium, and Zinc (2001).

^3^Food and Nutrition Board, Institute of Medicine, National Academy of Sciences. Dietary Reference Intakes for Energy, Carbohydrate, Fiber, Fat, Fatty Acids, Cholesterol, Protein, and Amino Acids (2002/2005); and Dietary Reference Intakes for Calcium and Vitamin D (2011).

P-values for nutrients intakes obtained from independent t test analysis and significance level set at 0.05.

The prevalence was 68% for the females and 74% in the males (p > 0.05, Mann-Whitney test). Females had higher prevalence of anaemia (41.5%) compared with males (21.8%), (p < 0.005, Mann-Whitney test). All females had CRP levels above the cut off (10.0ng/ml) in this study. The total prevalence of malaria parasitaemia among the children was (67.8%); females (66.2%) and males (69.2%). Hookworm infestation was 18.0% and was only observed among male participants. The mean caloric (energy) intakes of male participants (1018.61±261.27kcal) was higher than that of females (939.50±244.36kcal) even though the mean difference was not statistically significant (p>.05) (Table 2). No child had a Nutrient Adequacy Ratio (NAR) < 1 for dietary iron intake. Nine-percent of males and females had NAR < 1 for protein; 77% females and 72% males, respectively, had NAR < 1 for vitamin C.

Age was correlated with malaria parasitaemia (r = -.223; p = 0.035), haemoglobin concentration (r=.374; p = 0.001) and C-reactive protein levels (r = -.278; p = 0.007) (Table 3).

**Table 3:** Correlation between haematological, inflammation variables and age of study participants

	Haemoglobin	Ferritin	CRP	MP	Child age
**Haemoglobin**	1				
**Ferritin**	-.022	1			
**CRP**	-.285**	.288*	1		
**MP**	-.175	.005	.319*	1	
**Child age**	.374**	.140	-.278**	-.223*	1

* Correlation is significant at 0.05 level.

** Correlation is significant at 0.01 level.

CRP = C-reactive protein; MP = Malaria Parasitaemia.

Similarly C-reactive protein levels were found to be correlated with malaria parasitaemia (.319, p = 0.012), haemoglobin concentration (r = -.285; p = 0.006) and ferritin levels (r=.288; p = 0.026).

Child's sex and C-reactive protein levels were associated with anaemia in the multivariable analysis (Table 4).

**Table 4:** Factors associated with anaemia among the study participants

Factor	Odd Ratio	95% CI	P-value
**Child age**			
< 9years	0.805	0.26 – 2.54	.711
≥ 9 years	1.0	Reference	
**Gender**			
Males	0.172	0.06– 0.54	.002
Females	1.0	Reference	
**Ferritin concentration (ng/ml)**			
14.2 – 25.3	0.305	0.08 – 1.160	.081
≥ 25.4	1.0	Reference	
**Malaria/inflammation**			
Malaria absent	0.529	0.21 – 1.11	.170
Malaria present	1.0	Reference	
C-reactive protein level ≥ 10	.977	0.957 – 0.998	.031
ng/ml	1	Reference	
**C-reactive protein level.< 10**			
**ng/ml**			

Statistical significance p < 0.05, OR (Odds Ratio), Hosmer and Lemeshow test of significance (P = .192), Nagelkerke R^2^ = .259, adjusting for child age and C-reactive protein level.

Sex was significantly associated with anaemia. Males were less likely than females to be anaemic after adjusting for age and C-reactive protein levels (OR = 0.2, CI 0.1-0.5, p = 0.002). Ferritin concentration and presence of malaria parasitaemia were not associated with anaemia (p > 0.05).

## Discussion

The aim of this study was to assess the prevalence of anaemia and examine its associated factors among Ghanaian school children aged 6-12 years. We observed a high prevalence of anaemia, particularly among females, and low iron stores. The results are similar to findings from studies conducted in Ghana, Kenya, India and Bangladesh [11, 16–18] where multiple causes of anaemia were found to co-exist. We found that the prevalence of anaemia and low iron store among the children in the current study corroborates earlier studies [11, 23, 28] that anaemia is a public health issue in the West African sub region.

The prevalence of anaemia among the female participants was in excess of 40%, the level used to indicate a public health problem [22]. Previous studies have also found a higher prevalence of anaemia among females [29]. Caloric intake was slightly lower in females and Vitamin C, (known enhancer of iron bioavailability and absorption) inadequacy was also slightly higher in females. These factors may partially explain the higher rate of anaemia in females. The increased iron requirements related to rapid growth and development of females may also account for the difference in anaemia prevalence [30, 31]. The high prevalence of anaemia among females may have implications for their development [32].

Interestingly, the findings showed that both male and female participants consumed higher levels of iron than the recommended daily requirements [33]. This suggests that other factors such as the presence of iron absorption inhibitors such as phytic acid, tannins and polyphenols (not investigated in this study) in addition to infection (malaria parasitaemia), infestation (hookworm), and probably low bioavailability of non-haeme iron might have produced adverse nutritional effects among study participants. Few participants met the Estimated Average Requirement for Vitamin C [34], a known enhancer of non-haeme iron bioavailability [35–37] which may also explain the high prevalence of anaemia and low iron stores. Further research is warranted on factors that may affect iron bioavailability. Malaria parasitaemia destroys red blood cells and therefore its presence in the participants leads to low haemoglobin levels that puts infected children at higher risk of anaemia.

Hookworm infestation was only observed in males in the study. Since hookworm is a soil transmitted helminthes (human and animal faeces are major vehicles for spreading soil transmitted helminthes) it is probable that the males might have come into contact with hookworm-infested faecal matter through outdoor activities.

### Limitations of the study

There are several limitations to the present study that warrant acknowledgement. First, the study involved pupils (aged 6-12 years) from public schools not benefiting from Ghana School Feeding Programme (GSFP) at Adaklu-Kodzobi. Study findings may therefore not be generalized to out-of-school children and those in schools benefiting from the GSFP. Secondly, the use of 24-hour recall alone for nutrients intake assessment may not reflect the actual nutrient intake. Thirdly, due to the high prevalence of malaria coupled with high CRP concentrations, the cut-off point for serum ferritin concentration was raised to 30.0ng/ml instead of the normal cut off point of 12.0ng/ml to cater for the effect of infection or inflammation that would result in elevated ferritin levels. The few participants without malaria parasitaemia but with normal ferritin levels between 12.0ng/ml and 30.0ng/ml were considered as having low iron store which may have resulted in an over estimation of prevalence of low iron store among the study participants. Finally, the cross-sectional nature of the study means that we cannot make causal inferences.

## Conclusion

Study findings demonstrate a high prevalence of anaemia among children aged 6-12 years attending a school not benefiting from the Ghana School Feeding Programme. Females, in particular, were more likely to be anaemic and therefore vulnerable to adverse consequences of anaemia and iron deficiency. Study findings underscore the need for multi-pronged approaches that address both malaria control and nutrition in order to reduce anaemia among pupils.
